# The impact of trimethoprim-sulfamethoxazole as *Pneumocystis jiroveci* pneumonia prophylaxis on the occurrence of asymptomatic bacteriuria and urinary tract infections among renal allograft recipients: a retrospective before-after study

**DOI:** 10.1186/s12879-016-1432-3

**Published:** 2016-02-25

**Authors:** Ramandeep Singh, Frederike J. Bemelman, Caspar J. Hodiamont, Mirza M. Idu, Ineke J. M. ten Berge, Suzanne E. Geerlings

**Affiliations:** Department of Internal Medicine, Renal transplant Unit, Division of Nephrology, Academic Medical Center – University of Amsterdam, PO box 22660, 1100 DD Amsterdam, The Netherlands; Department of Medical Microbiology, Academic Medical Center – University of Amsterdam, PO box 22660, 1100 DD Amsterdam, The Netherlands; Department of Surgery, Division of Vascular Surgery, Academic Medical Center – University of Amsterdam, PO box 22660, 1100 DD Amsterdam, The Netherlands; Department of Internal Medicine, Division of Infectious Diseases, Academic Medical Center-University of Amsterdam, PO box 22660, 1100 DD Amsterdam, The Netherlands

**Keywords:** Asymptomatic bacteriuria, Urinary tract infections, Renal transplantation, Trimethoprim-sulfamethoxazole, *Pneumocystis jiroveci* pneumonia prophylaxis

## Abstract

**Background:**

The international guidelines recommend the administration of trimethoprim-sulfamethoxazole (TMP-SMX) as *Pneumocystis jiroveci* pneumonia (PJP) prophylaxis for six months after transplantation. The aim of this study is to evaluate the influence of TMP-SMX prophylaxis on the occurrence of asymptomatic bacteriuria (ASB) and urinary tract infections (UTIs) as cystitis and allograft pyelonephritis (AGPN) and its impact on the antimicrobial resistance pattern of causative microorganisms.

**Methods:**

We have conducted a retrospective before-after study in adult renal allograft recipients with one year follow-up after transplantation. We compared the (“after”) group that received TMP-SMX as PJP prophylaxis to the (“before”) group that did not receive it.

**Results:**

In total, 343 renal allograft recipients were analysed, of whom 212 (61.8 %) received TMP-SMX as PJP prophylaxis. In this study, 63 (18.4 %) did only develop ASB without UTI, 26 (7.6 %) developed cystitis and 43 (12.5 %) developed AGPN. The remaining 211 (61.5 %) renal allograft recipients did not develop any bacteriuria at all. Multivariable Cox proportional regression analysis indicated that TMP-SMX as PJP prophylaxis was not associated with reduced prevalence of ASB (Hazard ratio (HR) = 1.52, 95 % CI = 0.79–2.94, *p* = 0.213), nor with reduced incidence of cystitis (HR = 2.21, 95 % CI = 0.76–6.39, *p* = 0.144), nor AGPN (HR = 1.12, 95 % CI = 0.57–2.21, *p* = 0.751). Among the group receiving TMP-SMX as PJP prophylaxis there was a trend was observed in increase of both amoxicillin (86 % versus 70 %) and TMP-SMX (89 % versus 48 %) resistance which already appeared within the first 30 days after TMP-SMX exposure.

**Conclusions:**

Among renal allograft recipients, administration of TMP-SMX as PJP prophylaxis does not prevent ASB nor UTI, however it is associated with tendency towards increased amoxicillin and TMP-SMX resistance.

## Background

Bacteriuria which is the most common infectious complication after renal transplantation [1] is categorised in asymptomatic bacteriuria (ASB) and urinary tract infections (UTIs) as cystitis and allograft pyelonephritis (AGPN). The incidence of bacteriuria is the highest within the first three months after renal transplantation [2] and many risk factors for bacteriuria in general and for both ASB and UTI separately have been described in the medical literature [3]. Administration of low dose antibiotics as UTI prophylaxis is commonly implemented within different patient groups. For example, among non-pregnant women experiencing recurrent UTIs, low dose antimicrobial prophylaxis has been shown to be effective in preventing UTIs [4]. Renal allograft recipients also receive low dose antibiotics, which is trimethoprim-sulfamethoxazole (TMP-SMX) intended as *Pneumocystis jiroveci* pneumonia (PJP) prophylaxis for six months after transplantation as recommended by international guidelines [2, 5].

The aim of this study is to evaluate the influence of TMP-SMX intended as PJP prophylaxis on both the prevalence of ASB and incidence of UTIs among renal allograft recipients. In addition to this, we also evaluate the impact of TMP-SMX as PJP prophylaxis on the antimicrobial resistance pattern of the microorganisms causing these bacteriuria events.

## Methods

### Study design

Retrospective before-after study with adult renal allograft recipients. The administration of TMP-SMX as PJP prophylaxis was implemented in June 2007. The “before” group consisted of those renal allograft recipients that did not receive TMP-SMX and the “after” group consisted of those who did receive it. We compared the group that received TMP-SMX as PJP prophylaxis to the group that did not receive it. Renal allograft recipients transplanted between July 2005 (start of implementing external stented ureterocystostomy) and 2009 were analysed, with one year follow-up after transplantation for developing bacteriuria.

### Definitions

ASB was defined as a bacteriuria event (at least 10^5^ colony-forming units (CFU)/ml) without any clinical symptoms suggestive for UTI [2, 6]. UTI was categorised into cystitis and AGPN. Cystitis was defined as the presence of leukocyturia (≥10 leukocytes per high power field microscopy analysis), bacteriuria (≥ 10^4^ CFU/ml) with symptoms of the lower urinary tract (urgency, frequency and dysuria) without fever. AGPN was defined as the presence of leukocyturia, bacteriuria (≥10^4^ colony-forming units (CFU)/ml) and fever (>38.0 °C) [2] with exclusion of other infectious cause for fever.

Delayed graft function was defined as the requirement of dialysis within the first week after transplantation [7]. Acute rejection episode was diagnosed through a renal allograft biopsy.

Cytomegalovirus (CMV) disease was defined as a positive quantitative polymerase chain reaction (PCR) with end organ involvement resulting in fever, gastrointestinal disease, pneumonia, hepatitis nephritis and/or retinitis as described by the guidelines [8, 9].

### Renal allograft surgery procedures and medications

The renal allograft was positioned into the iliac fossa through an extraperitoneal approach. Cephamandole was routinely administered as peroperative antimicrobial prophylaxis. All renal allograft recipients received external stented ureterocystostomy with an 8 French catheter. This stent was inserted in the bladder through a suprapubic puncture and positioned in the pelvis of the renal allograft and was routinely removed after five days. The Foley catheter, which was inserted pre-operatively, was removed after seven days if urine leakage was excluded by cystography on that same day.

All renal allograft recipients receive triple immunosuppressive therapy which includes steroids combined with mycophenolate mofetil or mycophenolic acid and a calcineurin inhibitor, mostly tacrolimus but alternatively cyclosporine. If necessary, induction therapy with basiliximab was given pre-operatively according to the international guidelines [10].

Valganciclovir was provided for a period of six months after renal transplantation. Trimethoprim-sulfamethoxazole as PJP prophylaxis at a daily dose of 480 mg was implemented in June 2007. After transplantation, all renal allograft recipients received this prophylaxis for a period of six months. Recipients being allergic for, or having other contraindications for TMP-SMX did not receive TMP-SMX prophylaxis.

### Patient follow-up after transplantation

After discharge from the renal transplantation ward, all renal transplant recipients were frequently followed within our outpatient clinics. During the first three months after transplantation, all renal allograft recipients were seen twice per week on average. Hereafter, renal allograft recipients were seen once a month within the first year after transplantation.

Surveillance for bacteriuria occurred through screening for leukocyturia within the urine sediment. In case of leukocyturia, a urine culture was taken. A urine culture was also taken in case of fever or urinary tract symptoms. On average, every renal allograft recipient received 22 urine sediment analyses/urine cultures within the first year after transplantation. Only ASB episodes occurring within the first three months after transplantation were treated. Hereafter, ASB was not systematically treated since the international guideline does not give a consensus of management [2, 11].

### Susceptibility testing

Susceptibility testing was performed either using disk diffusion according to EUCAST criteria or using the VITEK2® system (BioMérieux, France). Interpretation of susceptibility results was performed using EUCAST breakpoints. Microorganisms were classified as “resistant” against a certain antimicrobial agent if the MIC reported by the VITEK2® system or the zone diameter using disk diffusion exceeded the breakpoint for susceptibility.

### Antimicrobial resistance pattern and TMP-SMX exposure

To evaluate the impact of TMP-SMX as PJP prophylaxis on the antimicrobial resistance pattern of the causative microorganisms, we determined the frequency of resistance to amoxicillin, TMP-SMX, amoxicillin-clavulanic acid, ciprofloxacin and nitrofurantoin among *E. coli* isolates according to TMP-SMX exposure. These *E. coli* isolates were subcategorised in three arbitrary time-frames according to the time between transplantation and urine culture. The “early” time-frame consisted of *E. coli* isolates cultured within the first 30 days after transplantation, those cultured between 31 and 180 days were categorised within the “intermediate” time-frame. The third time-frame consisted of *E. coli* isolates cultured between 181 and 365 days were categorised into “late” time-frame.

Each UTI was considered as a unique episode. Multiple ASB episodes in one renal allograft recipient were considered as unique in case of different genus of the cultured causative microorganism. In case of multiple ASB episodes with the same causative microorganism; these cultures were considered as unique if there was at least one negative urine culture between them.

### Statistical analysis

Statistical analysis was performed by using SPSS version 21 software (IBM SPSS Statistics for Windows, Version 21.0. Armonk, NY: IBM Corp.). Figures were made with GraphPad Prism version 5.00 for windows, GraphPad Software, San Diego California USA.

Continuous variables were expressed as the mean with its standard deviation in case of normal distribution. In case of non-normal distribution, the median and (25–75 %) interquartile range (IQR) were noted. In case of normal distribution, Student’s t-test was used to compare continuous variables between two groups. In case of non-normal distribution Mann Whitney-U test was used. Categorical variables were expressed as proportion (n) and percentage (%), and were compared using Chi-square test.

To determine the risk factors for ASB and UTI (cystitis or AGPN), we compared the group that did not develop any bacteriuria at all (reference group) to the group that developed respectively only ASB, cystitis or AGPN. These events were analysed separately with Cox proportional hazard model analysis.

To determine the hazard of developing respectively ASB or UTI according to TMP-SMX as PJP prophylaxis use, we first performed univariable Cox proportional hazard model in which ASB or UTI were the dependent variable and TMP-SMX the independent variable. In the first multivariable model we adjusted for the group differences stratified according to TMP-SMX use. In the second multivariable model we adjusted for both the group differences according to TMP-SMX use and also for variables significantly associated with developing respectively ASB or UTI obtained by the univariable COX proportional hazard model. The results of these analysis were reported as hazard ratio (HR) with its 95 % confidence interval (95 % CI). The hazards were proportional over time, we tested this by defining the two groups as a function over time variable, which we divided into two equal periods. A *p* value smaller than 0.05 was considered as statistically significant.

## Results

### Comparison of the group without and with TMP-SMX as PJP prophylaxis

Table 1 gives an overview of the entire group according to TMP-SMX as PJP prophylaxis use. In total, 343 renal allograft recipients with one-year follow-up were analysed, 212 (61.8 %) received TMP-SMX as PJP prophylaxis in the “after” group. In total 17 renal allograft recipients transplanted after June 2007 did not receive TMP-SMX as result of allergy/intolerance and were classified in the “no TMP-SMX” group.

**Table 1:** Comparison between the group without and with TMP-SMX as PJP prophylaxis

	Total	No TMP-SMX	TMP-SMX	*P* value
	*N* = 343 (100 %)	*N* = 131 (38.2 %)	*N* = 212 (61.8 %)	
Variables				
Age recipient	52 (40–61)	52 (40–59)	52 (39–61)	0.420
BMI recipient	25.2 +/−4.5	24.7 +/− 4.2	25.5 +/−4.6	0.128
Female gender	152 (44.3)	65 (49.6)	87 (41.0)	0.120
Diabetes Mellitus (a)	94 (27.4)	25 (19.1)	69 (32.5)	0.007
Age donor	50 (40–57)	49 (39–57)	51 (41–57)	0.232
Allograft from deceased donor	215 (62.7)	83 (63.4)	132 (62.3)	0.839
Delayed graft function	109 (31.8)	42 (32.1)	67 (31.6)	0.930
Acute rejection	80 (23.3)	31 (23.7)	49 (23.1)	0.925
Indwelling urological catheter (b)	53 (15.5)	10 (7.6)	43 (20.3)	0.002
First transplantation	294 (85.7)	113 (86.3)	181 (85.4)	0.821
CMV disease	26 (7.6)	13 (9.9)	13 (6.1)	0.197
Maintenance therapy				<0.001
Tacrolimus-MMF-steroids	205 (59.8)	49 (37.4)	156 (73.2)	
MMF-cyclosporine-steroids	38 (11.1)	28 (21.4)	10 (4.7)	
MA-cyclosporine-steroids	100 (29.2)	54 (41.2)	46 (21.7)	
Induction therapy				
Basiliximab	216 (63.0)	82 (62.6)	134 (63.2)	0.909
Primary renal disease				
Hypertension	88 (25.7)	23 (17.6)	65 (30.7)	
Cystic renal disease	49 (14.3)	18 (13.7)	31 (14.6)	
IgA nephropathy	28 (8.2)	15 (11.5)	13 (6.1)	
Diabetes	20 (5.8)	7 (5.3)	13 (6.1)	
Focal segmental glomerulosclerosis	27 (7.9)	11 (8.4)	16 (7.5)	
Reflux and anatomical abnormalities	25 (7.3)	11 (8.4)	14 (6.6)	
Glomerulonephritis	26 (7.6)	10 (7.6)	16 (7.5)	
Unknown origin	27 (7.9)	12 (9.2)	15 (7.1)	
Others	53 (15.5)	24 (18.3)	29 (13.7)	
Bacteriuria outcomes:				
No bacteriuria	211 (61.5)	94 (71.7)	117 (55.2)	0.002
Bacteriuria	132 (38.5)	37 (28.2)	95 (44.8)	
*Subtype of bacteriuria*				
- only ASB	63 (18.4)	17 (13.0)	46 (21.7)	
- cystitis	26 (7.6)	5 (3.8)	21 (9.9)	
- AGPN	43 (12.5)	15 (11.5)	28 (13.2)	

Continuous variables are depicted as mean with +/− standard deviation or as median with (25–75 %) interquartile range. Nominal variables are depicted as the total number analysed with its percentage (%). *AGPN* allograft pyelonephritis, *ASB* asymptomatic bacteriuria, *CI* confidence interval, *CMV* cytomegalovirus, *MA* mycophenolic acid, *MMF* mycophenolate mofetil, *TMP-SMX* trimethoprim-sulfamethoxazole

a: The variable “diabetes mellitus” includes type 1, type 2 diabetes and new onset of diabetes after transplantation (NODAT), irrespective of whether it was the primary disease which led to renal failure. b: The variable “Indwelling urological catheter” represents Foley catheter, nephrostomy catheter and intermittent self-catheterisation

There were three significant differences between the group without and with TMP-SMX as PJP prophylaxis; diabetes mellitus (*p* = 0.007), indwelling urological catheters (*p* = 0.002) and tacrolimus based immunosuppressive therapy (*p* < 0.001) were more prevalent in the group receiving TMP-SMX as PJP prophylaxis in comparison to the group without it.

### ASB and UTI rate according to TMP-SMX administration

Table 1 also displays the amount of bacteriuria according to TMP-SMX exposure. Within one year after transplantation, 211 (61.5 %) out of 343 renal allograft recipients did not develop any episode of bacteriuria at all, 63 (18.4 %) recipients developed only ASB, 26 (7.6) developed cystitis and 43 (12.5) developed AGPN. In comparison to the group without TMP-SMX as PJP prophylaxis, bacteriuria more prevalent among the group that did receive it (44.8 % versus 28.2 %, *p* = 0.002).

Within one year after transplantation, the cumulative amount of unique bacteriuria episodes was 316 within the entire study group; 79 episodes (25.2 %) occurred in the group without TMP-SMX prophylaxis and 236 episodes (74.9 %) occurred in the group with this prophylaxis.

### Risk factors for ASB and UTIs

Table 2 displays the univariable analysis of the variables associated with developing ASB, cystitis or AGPN respectively compared to the reference group that did not developed any bacteriuria at all. Risk factors for ASB were advanced age of the recipient (HR = 1.02, 95 % CI = 1.00–1.04, *p* = 0.029), female gender (HR = 2.01, 95 % CI = 1.22–3.33, *p* = 0.007) advanced age of the donor (HR = 1.03, 95 % CI = 1.01–1.05, *p* = 0.010) and indwelling urological catheters (HR = 12.94, 95 % CI = 7.76–21.57, *p* < 0.001).

**Table 2:** Univariable comparison between the group that did not develop any bacteriuria at all and the group that developed respectively only ASB, cystitis or AGPN

	No bacteriuria versus only ASB				No bacteriuria versus cystitis			No bacteriuria versus AGPN		
	No bacteriuria (REF) *N* = 211 (61.5 %)	Only ASB *N* = 63 (18.4 %)	Univariable analysis HR (95 % CI)	*P* value	Cystitis *N* = 26 (7.6 %)	Univariable analysis HR (95 % CI)	*P* value	AGPN *N* = 43 (12.5 %)	Univariable analysis HR (95 % CI)	*P* value
Variables										
TMP-SMX prophylaxis	117 (55.5)	46 (73.0)	2.03 (1.17–3.55)	0.012	21 (80.8)	3.12 (1.18–8.28)	0.022	28 (65.1)	1.46 (0.78–2.73)	0.239
Age of recipient	51 (38–59)	54 (46–64)	1.02 (1.00–1.04)	0.029	56 (44–60)	1.03 (1.00–1.06)	0.108	54 (38–62)	1.01 (0.99–1.04)	0.282
Female gender	85 (40.3)	38 (60.3)	2.01 (1.22–3.33)	0.007	13 (50.0)	1.43 (0.67–3.10)	0.357	16 (37.2)	1.18 (0.63–2.18)	0.610
Diabetes Mellitus (a)	49 (23.2)	19 (30.2)	1.37 (0.80–2.35)	0.250	13 (50.0)	1.13 (1.45–6.75)	0.004	13 (30.2)	1.39 (0.73–2.67)	0.320
BMI of recipient	25.0 +/−4.30	25.3 +/−4.35	1.02 (0.96–1.08)	0.576	27.0 +/−6.7	1.09 (1.01–1.17)	0.027	25.0 +/−3.9	1.00 (0.94–1.08)	0.932
Age of donor	49 (40–56)	53 (43–65)	1.03 (1.01–1.05)	0.010	51 (40–58)	1.00 (0.97–1.03)	0.738	48 (37–57)	1.00 (0.98–1.02)	0.992
Allograft from deceased donor	122 (57.8)	50 (79.4)	1.22 (0.73–2.03)	0.453	23 (88.5)	5.28 (1.59–17.60)	0.007	30 (69.8)	1.61 (0.84–3.09)	0.152
Delayed graft function	60 (28.4)	19 (30.2)	1.08 (0.63–1.84)	0.790	13 (50.0)	2.41 (1.12–5.21)	0.025	17 (39.5)	1.56 (0.85–2.87)	0.156
Acute rejection	43 (20.4)	19 (30.2)	1.56 (0.91–2.67)	0.109	8 (30.8)	1.64 (0.71–3.78)	0.244	10 (23.3)	1.12 (0.55–2.28)	0.751
Indwelling urological catheter (b)	5 (2.4)	28 (44.4)	12.94 (7.76–21.57)	<0.001	5 (19.2)	8.22 (3.09–21.90)	<0.001	15 (34.9)	11.24 (5.92–21.33)	<0.001
First transplantation	180 (85.3)	54 (85.7)	1.04 (0.52–2.11)	0.907	22 (84.6)	0.97 (0.34–2.83)	0.961	38 (88.4)	1.27 (0.50–3.22)	0.619
CMV disease	16 (7.6)	6 (9.5)	1.26 (0.54–2.92)	0.592	1 (3.8)	0.50 (0.07–3.66)	0.491	3 (7.0)	0.88 (0.28–2.87)	0.842
Maintenance therapy										
Tacrolimus + MMF + steroids	122 (57.8)	41 (65.1)	1.00		18 (69.2)	1.00		24 (55.8)	1.00	
MMF + cyclosporine + steroids	22 (10.4)	8 (12.7)	1.02 (0.48–2.19)	0.851	2 (7.7)	0.65 (0.15–2.81)	0.567	6 (14.0)	1.30 (0.53–3.19)	0.563
MA + cyclosporine + steroids	67 (31.8)	14 (22.2)	0.65 (0.35–1.19)	0.164	6 (23.1)	0.63 (0.25–1.57)	0.319	13 (30.2)	0.97 (0.49–1.90)	0.919
Induction therapy										
Basiliximab	134 (63.5)	39 (61.9)	0.94 (0.56–1.56)	0.805	43 (52.3)	0.58 (0.27–1.24)	0.159	30 (69.8)	1.28 (0.67–2.46)	0.455

Continuous variables are depicted as mean with +/− standard deviation or as median with (25–75 %) interquartile range. Nominal variables are depicted as the total number analysed with its percentage (%). *AGPN* allograft pyelonephritis, *ASB* asymptomatic bacteriuria, *CI* confidence interval, *CMV* cytomegalovirus, *MA* mycophenolic acid, *MMF* mycophenolate mofetil, *NA* not applicable, *HR* hazard ratio, *REF* reference, *TMP-SMX* trimethoprim-sulfamethoxazole

a: The variable “diabetes mellitus” includes type 1, type 2 diabetes and new onset of diabetes after transplantation (NODAT), irrespective of whether it was the primary disease which led to renal failure

b: The variable “Indwelling urological catheter” represents Foley catheter, nephrostomy catheter and intermittent self-catheterisation

Risk factors for cystitis were diabetes mellitus (HR = 1.13, 95 % CI = 1.45–6.75, *p* = 0.004), BMI of recipient (HR = 1.09, 95 % CI = 1.01–1.17, *p* = 0.027), receiving a renal allograft obtained from deceased donor (HR = 5.28, 95 % CI = 1.59–17.60, *p* = 0.007), delayed graft function (HR = 2.41, 95 % CI = 1.12–5.21, *p* = 0.025) and indwelling urological catheters (HR = 8.22, 95 % CI = 3.09–21.90, *p* < 0.001). The only identified risk factor for AGPN was the presence of an indwelling urological catheter (HR = 11.24, 95 % CI = 5.92–21.33, *P* < 0.001).

### Influence of TMP-SMX as PJP prophylaxis on the occurrence of ASB and UTIs

Since the group without and with TMP-SMX as PJP prophylaxis were not comparable to each other on the variables diabetes mellitus, the presence of urological catheters and the subtype of the immunosuppressive therapy (Table 1), we performed multivariable Cox proportional hazard regression analysis in which we also adjusted for these three variables (Tables 3, 4 and 5).

**Table 3:** Univariable and multivariable Cox regression analysis for developing ASB within one year after transplantation according to TMP-SMX prophylaxis administration

	Outcome ASB	*P* value
	HR (95 % CI)	
TMP-SMX: univariable model (a)	2.03 (1.17–3.55)	0.012
TMP-SMX: first multivariable model (b)	1.18 (0.63–2.20)	0.600
TMP-SMX: second multivariable model (c)	1.52 (0.79–2.94)	0.213

a: Univariable model: adjusted for only TMP-SMX use

b: First multivariable analysis: adjusted for diabetes mellitus, subtype of immunosuppressive therapy and indwelling urological catheters

c: Second multivariable analysis: adjusted for diabetes mellitus, subtype of immunosuppressive therapy, indwelling urological catheters, age of recipient and donor and female gender

**Table 4:** Univariable and multivariable Cox regression analysis for developing cystitis within one year after transplantation according to TMP-SMX prophylaxis administration

	Outcome cystitis	*P* value
	HR (95 % CI)	
TMP-SMX: univariable model (a)	3.12 (1.18–8.28)	0.022
TMP-SMX: first multivariable model (b)	2.29 (0.79–6.67)	0.127
TMP-MSX: second multivariable model (c)	2.21 (0.76–6.39)	0.144

a: Univariable model: adjusted for only TMP-SMX use

b: First multivariable analysis: adjusted for diabetes mellitus, subtype of immunosuppressive therapy and indwelling urological catheters

c: Second multivariable analysis: adjusted for diabetes mellitus subtype of immunosuppressive therapy, indwelling urological catheters, BMI of recipient, type of renal allograft (obtained from a deceased versus living donor) and delayed graft function

**Table 5:** Univariable and multivariable Cox regression analysis for developing AGPN within one year after transplantation according to TMP-SMX prophylaxis administration

	Outcome AGPN	*P* value
	HR (95 % CI)	
TMP-SMX: univariable model (a)	1.46 (0.78–2.73)	0.239
TMP-SMX: first multivariable model (b)	1.12 (0.57–2.21)	0.751

a: Univariable model: adjusted for only TMP-SMX use

b: First multivariable analysis: adjusted for diabetes mellitus, subtype of immunosuppressive therapy and indwelling urological catheters. Since indwelling urological catheters were the only risk factors for AGPN no second multivariable analysis has been performed

In the univariable Cox proportional hazard regression analysis TMP-SMX was associated with developing ASB (HR = 2.03, 95 % CI = 1.17–3.55, *p* = 0.012) as shown in Table 3. After correcting for both the group differences according to TMP-SMX prophylaxis administration and for univariable risk factors for ASB (Table 3), TMP-SMX was not associated with reduced occurrence of ASB (HR = 1.52, 95 % CI = 0.79–2.94, *p* = 0.213).

In the univariable Cox proportional regression analysis, TMP-SMX was associated with developing cystitis (HR = 3.12, 95 % CI = 1.18–8.28, *p* = 0.022), however after adjustment for both the group differences according to TMP-SMX prophylaxis administration and univariable risk factors for developing cystitis (Table 4), TMP-SMX did not prevent cystitis (HR = 2.21, 95 % CI = 0.76–6.39, *p* = 0.144).

Administration of TMP-SMX as PJP prophylaxis was not associated with reduced incidence of AGPN in both univariable model (HR = 1.46, 95 % CI = 1.78–2.73, *p* = 0.239) nor in the adjusted model (HR = 1.12, 95 % CI = 0.57–2.21, *p* = 0.751) (Table 5).

**Fig. 1:**
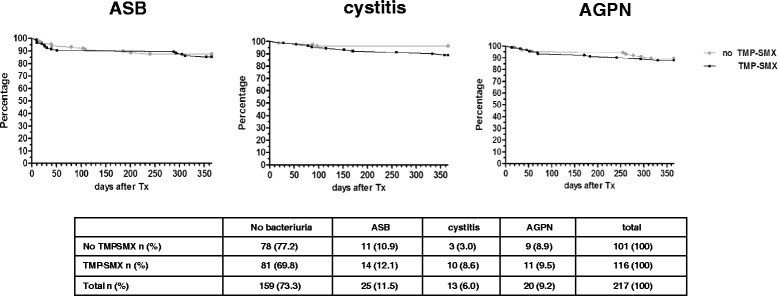
Analysis of the subgroup without diabetes nor any urological catheters in situ (*n* = 217). Kaplan Meijer curves for developing ASB, cystitis or AGPN according to TMP-SMX administration as PJP prophylaxis

To illustrate this, we additionally analysed the subgroup that did not develop diabetes, and never had indwelling urological catheter in situ (*n* = 217). For this subgroup, time between transplantation and respectively ASB, cystitis and AGPN is displayed in Fig. 1.

### Causative microorganisms and antimicrobial resistance pattern

In total 315 unique bacteriuria episodes were identified within one year after transplantation. The majority of the causative microorganisms of the bacteriuria events were by *Escherichia coli* (*E. coli*) and *Enterococcus* spp. (Table 6).

**Table 6:** Causative microorganisms of the unique bacteriuria episode according to TMP-SMX prophylaxis administration

	Entire cohort	No TMP-SMX	TMP-SMX
	*N* = 315 (100 %)	*N* = 79 (25.2 %)	*N* = 236 (74.9 %)
*Escherichia coli*	128 (40.6)	31 (39.2)	97 (41.1)
*Enterococcus* spp.	70 (22.2)	18 (22.8)	52 (22.0)
*Enterobacter* spp.	15 (4.8)	7 (8.9)	8 (3.4)
*Klebsiella* spp.	20 (6.3)	2 (2.5)	18 (7.6)
*Proteus* spp.	12 (3.8)	6 (7.6)	6 (2.6)
*Pseudomonas spp*.	38 (12.1)	7 (8.9)	31 (13.1)
Others	32 (10.2)	8 (10.1)	24 (10.2)

**Fig. 2:**
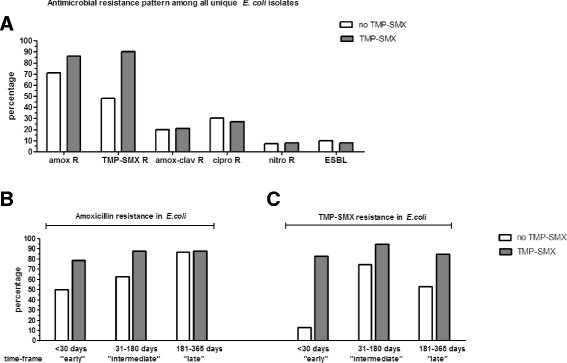
**a**. Antimicrobial resistance pattern of all *Escherichia coli* (*E. coli*) isolates (*n* = 128) cultured within one year after transplantation according to exposure to TMP-SMX as PJP prophylaxis. amox = amoxicillin. amox-clav = amoxicillin-clavulanic acid. cipro = ciprofloxacin. nitro = nitrofurantoin. TMP-SMX = trimethoprim-sulfamethoxazole. ESBL = Extended Spectrum beta-Lactamase. R = resistant. **b** and **c** Amoxicillin and TMP-SMX resistance among *E. coli* according to exposure to TMP-SMX as PJP prophylaxis. *E.coli.* isolates are categorised in three time groups. First group consists of *E. coli* isolates cultured within 30 days after transplantation (“early” time-frame). The second group consists of *E. coli* isolates cultured within 31-180 days after transplantation (intermediate time-frame). The third group consists of *E. coli* isolates cultured after 181 till 365 days after transplantation (“late” time-frame)

Figure 2a displays the percentage of resistance for the five antibiotics among *E. coli* isolates. Among the *E. coli* isolates cultured from the group that received TMP-SMX as PJP prophylaxis, there was a tendency towards higher resistance rate for both amoxicillin (86 % versus 70 %) and TMP-SMX (89 % versus 48 %) compared to the group that did not receive it. No differences in resistance rates of amoxicillin-clavulanic acid, ciprofloxacin or nitrofurantoin were observed.

To evaluate the change in amoxicillin and TMP-SMX resistance in course of time after transplantation, we compared the percentage of resistance against these two antibiotics among *E. coli* isolates cultured within the first 30 days after transplantation (“early” time-frame), between 31 and 180 days (“intermediate” time-frame) and finally within 181–365 days (“late” time-frame) after transplantation (Fig. 2b and c). Among the group with TMP-SMX as PJP prophylaxis, the *E. coli* isolates cultured within the first 30 days after transplantation, had a higher resistance rate to amoxicillin (80 % versus 50 %) and TMP-SMX (83 % versus 13 %), in comparison to *E. coli* isolates within the same time frame but without TMP-SMX exposure.

## Discussion

We were particularly interested whether TMP-SMX as PJP prophylaxis had any influence on the occurrence of ASB and UTI after renal transplantation. The administration of TMP-SMX as PJP prophylaxis was not associated with lower ASB prevalence nor lower UTI incidence. Furthermore, after TMP-SMX exposure, a tendency towards increased amoxicillin and TMP-SMX resistance was observed.

In this study, we observed that 18.4 % developed only ASB and 20.1 % developed UTI; of which 7.6 % developed cystitis and 12.5 % AGPN. In the medical literature, the incidence of bacteriuria shows a great variety as result of large differences in the used diagnostic criteria and frequency of routine urine culture testing [1, 12] The prevalence of ASB ranges between 17 % [13] and 40 % [14] within the first year after transplantation. Cystitis incidence has been reported at 12.2 % during three year follow-up [15] while the AGPN incidence ranges between 13 % [16] and 16.5 % [17] during four to ten year follow-up time.

Compared to the group without TMP-SMX as PJP prophylaxis, the group receiving TMP-SMX prophylaxis had significantly more bacteriuria as result of higher prevalence of diabetes and indwelling urological catheters. Increased diabetes prevalence in the group receiving TMP-SMX prophylaxis can most likely be explained by the more frequently administration of tacrolimus, which has decreased insulin sensitivity as a common side effect [18]. In addition to this, indwelling urological catheters are a great risk factor for bacteriuria; it has been estimated that there is a daily risk of 5 % for developing bacteriuria after catheterisation and approximately 70 % of catheterised patients develop bacteriuria after 14 days [19, 20]. As result of these three differences, we performed multivariable Cox regression analysis in which we adjusted for these three variables and for the variables associated with developing respectively ASB, cystitis or AGPN. Within these multivariable models, administration of TMP-SMX as PJP prophylaxis was not associated with reduced occurrence of ASB, cystitis or AGPN. Comparable outcome has been observed by another research group which reported that the use of one double-strength tablet of TMP-SMX three times a week did not prevent UTI defined as cystitis and pyelonephritis [21]. However, another study [22] demonstrated that the addition of 30-day of ciprofloxacin to TMP-SMX prophylaxis lowered the incidence of UTI in comparison to TMP-SMX prophylaxis alone. Interestingly, no difference was observed in the incidence of pyelonephritis.

The observation that TMP-SMX does not prevent ASB nor UTIs could likely be explained by the great potency of gram negative bacteria for developing TMP-SMX resistance after exposure to it. Indeed, it has been shown that TMP-SMX exposure results in TMP-SMX resistance in *E. coli*, which is even observed after one month of TMP-SMX administration [23].

The first clinical trials evaluating the effect of antimicrobial prophylaxis on the incidence of bacteriuria among renal allograft recipients were performed in late eighties and early nineties of the last millennium [24–26]. In these studies, TMP-SMX was used as prophylaxis. The inter-variability of TMP-SMX dose between these studies was large and ranged between 480 mg once a day to 960 mg twice a day. Also the duration of antimicrobial prophylaxis administration differed. A systematic review [27] which also included these studies concluded that antimicrobial prophylaxis indeed reduces bacteriuria and bacteraemia. However the analysed original intervention studies did not mention the clinical symptoms associated with bacteriuria. Therefore in these studies, it is difficult to determine whether TMP-SMX at prophylactic dose prevents ASB or UTI, or possibly both.

The second aim of our study was to evaluate the impact of TMP-SMX as PJP prophylaxis on the antimicrobial resistance pattern of the causative microorganisms. Among *E. coli* isolates cultured within the first 30 days after transplantation, there was an increase in both amoxicillin and TMP-SMX resistance after TMP-SMX exposure. This co-resistance of TMP-SMX with amoxicillin has also been described in other studies [23, 28] and is a common finding, since the resistance genes of these antimicrobials are located on the same plasmid [28–30]. However as a limitation, our study was not powered to observe statistical significant differences in antimicrobial resistance according to TMP-SMX exposure, therefore no statistical tests were performed in this analysis.

We also observed that the difference in amoxicillin resistance with and without TMP-SMX did not differ anymore among *E. coli* isolates cultured between 181 and 365 days after renal transplantation reflecting the antimicrobial resistance drift, since the likelihood of exposure to antibiotics increases in course of time. Indeed, excessive exposure to antibiotics is the greatest contributing factor for developing antimicrobial resistance [31]. However, among the *E. coli* isolates cultured between 181 and 365 days after transplantation, there was still a difference noticeable in TMP-SMX resistance with and without TMP-SMX exposure, indicating that TMP-SMX resistance developed after subsequent six months of TMP-SMX exposure might be able to persist for a longer period of time after discontinuation.

In this study we analysed renal allograft recipients transplanted between 2005 and 2009; a possibility exist that the increase of amoxicillin and TMP-SMX resistance according to TMP-SMX exposure could be influenced by an increase in antimicrobial resistance rate in course of time (background resistance). To evaluate this, we compared our data about the increase in amoxicillin and TMP-SMX resistance among *E. coli* isolates within this study, to those *E. coli* isolates cultured within unselected hospital departments across the Netherlands between 2005 and 2009. Within this time span, amoxicillin resistance rate among *E. coli* steadily increased from 44 to 48 % [32]. The background TMP-SMX resistance did not increase above 30 % in 2009 [32]. This increase in background antimicrobial resistance is smaller than the increase in antimicrobial resistance observed in our study population, which could be attributed to the six months exposure to TMP-SMX as PJP prophylaxis.

The unique aspect of this study is the comparison of a relative large group of renal allograft recipients who received TMP-SMX intended as PJP prophylaxis to a group who did not receive it. The limitations of our study are related to the retrospective and non-interventional setup. In retrospective studies, the determination of ASB prevalence is less accurate than determining UTI incidence because UTI is diagnosed by the presence of predefined clinical UTI symptoms accompanied by bacteriuria, while the rate of ASB is heavily influenced by the frequency of routine urine culturing [33]. Therefore, the likelihood for ASB detection increases by frequent urine culturing. To exclude potential bias caused by the frequency of testing, we analysed each ASB patient only once to determine risk factors for ASB. Another limitation of this study may be the policy of not systematically treating ASB episodes occurring three months after transplantation. Due to the retrospective setup, it is difficult to determine which circumstances resulted in the decision to treat a certain ASB episode occurring after three months transplantation.

## Conclusions

Our retrospective before-after study indicates that TMP-SMX prophylaxis does not have an additional bacteriuria preventive property besides PJP prevention. However it may have a certain contribution to an increase in amoxicillin and TMP-SMX resistance. Despite great advances made in renal transplantation medicine, there is still a need for effective UTI preventive measures among renal allograft recipients.

### Ethical consent

Due to the retrospective study setup, ethics approval was waived by the Medical Ethics Review Committee of the Academic Medical Center (Amsterdam, the Netherlands), where this study was performed.

### Data availability

The data analysed in this study can be accessed by sending a request to the corresponding author.

## Abbreviations

AGPN: allograft pyelonephritis
ASB: asymptomatic bacteriuria
CFU: colony forming unit
CI: confidence interval
CMV: cytomegalovirus
E. coli: *Escherichia coli*
HR: hazard ratio
PJP: *Pneumocystis jiroveci* pneumonia
TMP-SMX: trimethoprim-sulfamethoxazole
Tx: transplantation
UTI: urinary tract infection

## References

[1] Alangaden G (2007). Urinary tract infections in renal transplant recipients. Curr Infect Dis Rep.

[2] Parasuraman R, Julian K (2013). Urinary tract infections in solid organ transplantation. Am J Transplant.

[3] Singh R, Geerlings SE, Bemelman FJ (2015). Asymptomatic bacteriuria and urinary tract infections among renal allograft recipients. Curr Opin Infect Dis.

[4] Albert X, Huertas I, Pereiro, II, Sanfelix J, Gosalbes V, Perrota C. Antibiotics for preventing recurrent urinary tract infection in non-pregnant women. Cochrane Database Syst Rev. 2004(3):Cd001209. 10.1002/14651858.CD001209.pub2 PMC7032641 15266443

[5] Kasiske BL, Zeier MG, Chapman JR, Craig JC, Ekberg H, Garvey CA, Green MD, Jha V, Josephson MA, Kiberd BA et al. KDIGO clinical practice guideline for the care of kidney transplant recipients: a summary. Kidney Int. 2010;77(4):299–311. 10.1038/ki.2009.377 19847156

[6] Nicolle LE, Bradley S, Colgan R, Rice JC, Schaeffer A, Hooton TM (2005). Infectious Diseases Society of America guidelines for the diagnosis and treatment of asymptomatic bacteriuria in adults. Clin Infect Dis.

[7] Halloran PF, Hunsicker LG (2001). Delayed graft function: state of the art, November 10–11, 2000. Summit meeting, Scottsdale, Arizona, USA. Am J Transplant.

[8] Ljungman P, Griffiths P, Paya C (2002). Definitions of cytomegalovirus infection and disease in transplant recipients. Clin Infect Dis.

[9] Preiksaitis JK, Brennan DC, Fishman J, Allen U (2005). Canadian society of transplantation consensus workshop on cytomegalovirus management in solid organ transplantation final report. Am J Transplant.

[10] Kasiske BL, Zeier MG, Craig JC, Ekberg H, Garvey CA, Green MD, et al. KDIGO clinical practice guideline for the care of kidney transplant recipients. Am J Transplant. 2009;9(Suppl 3):S1–155. 10.1111/j.1600-6143.2009.02834.x 19845597

[11] Rice JC, Safdar N (2009). Urinary tract infections in solid organ transplant recipients. Am J Transplant.

[12] Saemann M, Horl WH (2008). Urinary tract infection in renal transplant recipients. Eur J Clin Investig.

[13] Green H, Rahamimov R, Goldberg E, Leibovici L, Gafter U, Bishara J, Mor E, Paul M. Consequences of treated versus untreated asymptomatic bacteriuria in the first year following kidney transplantation: retrospective observational study. Eur J Clin Microbiol Infect Dis. 2013;32(1):127–31. 10.1007/s10096-012-1727-2 22918514

[14] Golebiewska JE, Debska-Slizien A, Rutkowski B (2014). Treated asymptomatic bacteriuria during first year after renal transplantation. Transpl Infect Dis.

[15] Fiorante S, Fernandez-Ruiz M, Lopez-Medrano F, Lizasoain M, Lalueza A, Morales JM, San-Juan R, Andres A, Otero JR, Aguado JM . Acute graft pyelonephritis in renal transplant recipients: incidence, risk factors and long-term outcome. Nephrol Dial Transplant. 2011;26(3):1065–73. 10.1093/ndt/gfq531 20805254

[16] Giral M, Pascuariello G, Karam G, Hourmant M, Cantarovich D, Dantal J, Blancho G, Coupel S, Josien R, Daguin P et al. Acute graft pyelonephritis and long-term kidney allograft outcome. Kidney Int. 2002;61(5):1880–6. 10.1046/j.1523-1755.2002.00323.x 11967040

[17] Kamath NS, John GT, Neelakantan N, Kirubakaran MG, Jacob CK (2006). Acute graft pyelonephritis following renal transplantation. Transpl Infect Dis.

[18] Chen QJ, Li J, Zuo SR, Zhang YP, Jia SJ, Yuan H, Liu SK, Cheng K, Ming YZ, Zuo XC, et al. Tacrolimus decreases insulin sensitivity without reducing fasting insulin concentration: a 2-year follow-up study in kidney transplant recipients. Ren Fail. 2015;1–6. 10.3109/0886022X.2015.1007833 25644968

[19] Pickard R, Lam T, Maclennan G, Starr K, Kilonzo M, McPherson G, Gillies K, McDonald A, Walton K, Buckley B, et al. Types of urethral catheter for reducing symptomatic urinary tract infections in hospitalised adults requiring short-term catheterisation: multicentre randomised controlled trial and economic evaluation of antimicrobial- and antiseptic-impregnated urethral catheters (the CATHETER trial). Health Technol Assess (Winchester, England). 2012;16(47):1–197. 10.3310/hta16470 23199586

[20] Haley RW, Hooton TM, Culver DH, Stanley RC, Emori TG, Hardison CD, Quade D, Shachtman RH, Schaberg DR, Shah BV, et al. Nosocomial infections in U.S. hospitals, 1975–1976: estimated frequency by selected characteristics of patients. Am J Med. 1981;70(4):947–59. 10.1016/0002-9343(81)90561-1 6938129

[21] Vidal E, Torre-Cisneros J, Blanes M, Montejo M, Cervera C, Aguado JM, Len O, Carratala J, Cordero E, Bou G, et al. Bacterial urinary tract infection after solid organ transplantation in the RESITRA cohort. Transpl Infect Dis. 2012;14(6):595–603. 10.1111/j.1399-3062.2012.00744.x 22650416

[22] Wojciechowski D, Chandran S (2013). Effect of ciprofloxacin combined with sulfamethoxazole-trimethoprim prophylaxis on the incidence of urinary tract infections after kidney transplantation. Transplantation.

[23] Beerepoot MA, ter Riet G, Nys S, van der Wal WM, de Borgie CA, de Reijke TM (2012). Lactobacilli vs antibiotics to prevent urinary tract infections: a randomized, double-blind, noninferiority trial in postmenopausal women. Arch Intern Med.

[24] Fox BC, Sollinger HW, Belzer FO, Maki DG (1990). A prospective, randomized, double-blind study of trimethoprim-sulfamethoxazole for prophylaxis of infection in renal transplantation: clinical efficacy, absorption of trimethoprim-sulfamethoxazole, effects on the microflora, and the cost-benefit of prophylaxis. Am J Med.

[25] Maki DG, Fox BC, Kuntz J, Sollinger HW, Belzer FO (1992). A prospective, randomized, double-blind study of trimethoprim-sulfamethoxazole for prophylaxis of infection in renal transplantation. Side effects of trimethoprim-sulfamethoxazole, interaction with cyclosporine. J Lab Clin Med.

[26] Tolkoff-Rubin NE, Cosimi AB, Russell PS, Rubin RH (1982). A controlled study of trimethoprim-sulfamethoxazole prophylaxis of urinary tract infection in renal transplant recipients. Rev Infect Dis.

[27] Green H, Rahamimov R, Gafter U, Leibovitci L, Paul M (2011). Antibiotic prophylaxis for urinary tract infections in renal transplant recipients: a systematic review and meta-analysis. Transpl Infect Dis.

[28] den Heijer CD, Beerepoot MA, Prins JM, Geerlings SE, Stobberingh EE (2012). Determinants of antimicrobial resistance in Escherichia coli strains isolated from faeces and urine of women with recurrent urinary tract infections. PLoS One.

[29] Kahlmeter G, Menday P (2003). Cross-resistance and associated resistance in 2478 Escherichia coli isolates from the Pan-European ECO.SENS Project surveying the antimicrobial susceptibility of pathogens from uncomplicated urinary tract infections. J Antimicrob Chemother.

[30] Amyes SG (1989). The success of plasmid-encoded resistance genes in clinical bacteria. An examination of plasmid-mediated ampicillin and trimethoprim resistance genes and their resistance mechanisms. J Med Microbiol.

[31] Rao GG (1998). Risk factors for the spread of antibiotic-resistant bacteria. Drugs.

[32] Hoogkamp-Korstanje JAA, Mouton JW, Sande-Bruinsma N (2011). NETHMAP 2011 Consumption of antimicrobial agents and antimicrobial resistance among medically important bacteria in the Netherlands.

[33] Mitra S, Alangaden GJ (2011). Recurrent urinary tract infections in kidney transplant recipients. Curr Infect Dis Rep.

